# Entropy Is Not Extensive

**DOI:** 10.3390/e28060631

**Published:** 2026-06-03

**Authors:** Chris Jeynes, Michael C. Parker

**Affiliations:** 1 Independent Researcher, Tredegar NP22 4LP, UK; 2 School of Computer Science and Electrical Engineering, University of Essex, Colchester CO4 3SQ, UK

**Keywords:** emergence, mereology, statistical mechanics, action, QGT

## Abstract

The Gibbs Paradox (concerning the entropy of mixing and entropic extensivity) was explored in depth by Edwin Jaynes (1992). We take up Jaynes’ treatment, considering the special cases for which entropy is (approximately) extensive, and the general case in which it is not. We also explore the Holographic Principle which (strictly speaking) excludes the extensivity of entropy. The formalism of Quantitative Geometrical Thermodynamics shows that, being isomorphic to energy, it is entropy production (not entropy) that is extensive. As a corollary, Shannon information is also not extensive, although information production is extensive.

## 1. Introduction

Everyone knows that entropy is extensive: all the textbooks say so! For example, Table 1 shows Table 1.1 of C. J. Adkins’ classical textbook *Equilibrium Thermodynamics* (1968) [1], which explicitly represents *entropy* as extensive, without comment. But here we will show that this is ***not*** the case in general.

Edwin Jaynes said many times in different ways that people still really do not understand thermodynamics, and the issue of whether or not entropy is extensive is a central example. His understanding that “*the entropy of the whole is often less than the sum of the entropy of the parts*” (1992 [2]; clearly inimical to entropic extensivity) is therefore very timely today.

However, Richard Tolman (1917 [3]) has authoritatively expressed the conventional wisdom: that entropy ***is*** extensive, because “*the entropy of a system is the sum of the entropies of its parts*”. We will show that in fact this is ***not*** true in general: it is clearly not true if the parts are not independent; Tolman acknowledges that, although “*classification [between intensive/extensive] is usually simple*”, such classification may not be “*perfectly unambiguous*” (he also “explains” entropy as “*degree of run-downness*”, which we would not regard today as sufficiently constructive given the strong geometric properties that are now also assigned to entropy). Erwin Schrödinger (in his seminal *What is Life?*, 1944 [4]) was the first to point out that living organisms are necessarily dissipative structures, and Ilya Prigogine’s emphasis on *entropy production* in generating ordered systems was recognised with the 1977 Nobel Prize in Chemistry, for contributions to “*the theory of dissipative structures*”. In fact, it is the entropy production (the rate of change of entropy) which we will find to be unambiguously extensive. We should mention that there is also an important variational principle associated with entropy production: the “*maximum entropy production principle*”, usefully reviewed recently by Martyushev (2021 [5]).

The “*Gibbs’ Paradox*” (which is “*about the entropy of mixing and the logically inseparable topics of reversibility and the extensive property of entropy*”) was treated at length by Edwin Jaynes (1992, [2]), who pointed out that Willard Gibbs gave a correct analysis in 1875 [6] which was not included in his influential 1902 [7] monograph. The issue is that if two dissimilar gases are mixed the entropy rises, but the classical treatment appears not to distinguish the case when the gases are in fact identical (and the entropy should therefore not rise since nothing discernible happens: the quantum treatment recognises that when particles are indistinguishable the case is different).

However, Gibbs understood (and disposed of) this problem perfectly well in 1875, going “*straight to the heart of the matter as a simple technical detail*” (Jaynes, [2]). Unfortunately, these technical details remain poorly grasped even today, with much confusion remaining, partly due to a false belief that *statistical mechanics* is “more fundamental” than *thermodynamics*. But Michael te Vrügt et al. [8] have shown that it is thermodynamics that is fundamental (on which see Section 6). The Second Law is well recognised to be foundational, as Arthur Eddington famously pointed out (1928 [9] p. 74): “… *if your theory is found to be against the second law of thermodynamics I can give you no hope; there is nothing for it but to collapse in deepest humiliation*”.

Jaynes [2] shows that “*phenomenological thermodynamics*, *classical statistics*, *and quantum statistics are all in just the same logical position with regard to extensivity of entropy; they are silent on the issue*, *neither requiring it nor forbidding it*.” We shall consider simple cases for which entropy is (approximately) extensive, but also describe the *general case* where it is *not*.

Jaynes [2] draws attention to our central point: “*Gibbs … had perceived that*, *when two systems interact*, *only the entropy of the whole is meaningful … it is the entropy of the whole that contains full thermodynamic information. This reminds us of Gibbs’ famous remark*, *made in a supposedly (but perhaps not really) different context: ‘The whole is simpler than the sum of its parts.’ … For [Gibbs]*, *entropy is not a local property of the subsystems; it is a ‘global’ property like the Lagrangian of a mechanical system;* i.e., *it presides over the whole and determines*, *by its variational properties*, *all the conditions of equilibrium—just as the Lagrangian presides over all of mechanics and determines*, *by its variational properties*, *all the equations of motion*”. We can appreciate that lurking beneath Jaynes’ statement is the Holographic Principle, important for us with its “holistic” (non-local) properties which are therefore also clearly global (and non-extensive) in nature. In the variational Equation (3) below, we explicitly introduce the entropic Lagrangian that analytically represents the kind of global functionality sought by Jaynes.

However, Jaynes never recognised several key concepts in the non-extensive entropy of thermodynamics, including the fundamental ideas Alfréd Rényi and Terrill Hill introduced (in the early 1960s, when Jaynes was developing his own work; see Section 6).

Robert Swendson (2008 [10]), taking a different point of view, also discusses Gibbs’ Paradox. He elegantly demonstrates that an extensive entropy can be defined as a special case based on the statistics of distinguishable particles. That is, he proves that the traditional definition of the entropy in terms of a volume in phase space is indeed non-extensive, but that a definition of the entropy relying on Boltzmann’s treatment of macroscopic state probabilities (which appears to be similar to Jaynes’) can be shown to overcome the Paradox and is found to be extensive (although Swendson ignores Jaynes’ discussion of extensivity; see Section 3). However, it is clear that Swendsen is inclined to assume that entropy is extensive in general, even to the degree that he is tempted to make it a postulate (his “optional” postulate 5 [10]); yet, he then states that “*This postulate is not generally true*” and makes it optional. That is to say, it is always possible to consider special cases where entropy is found to exhibit extensivity; but, critically, this is *not* the general case! Entropy is not intrinsically extensive, which is our point here.

We will take a different point of view again, relying on the Quantitative Geometrical Thermodynamics (QGT) formalism, which independently generates the Holographic Principle (see Section 4). This is to contrast with the authoritative studies of Swendsen (2017 [11]), who also concisely discusses the necessity of relaxing the requirement of extensivity for various definitions of entropy (including the Boltzmann entropy, the Gibbs entropy, and the canonical and grand-canonical entropies). However, it is also clear that Swendsen is only considering the “traditional” forms of entropy related to 19th-century thermodynamics and statistical mechanics, and overlooks the *geometrical* form of entropy which is a more modern innovation, and which is in general not extensive, but which is also increasingly being found to be fundamental to our understanding of many key features of the Universe.

## 2. Simple Systems

It is very easy to show why entropy is (thought to be) extensive; the standard classical thermodynamics point of view starts from the First Law (and also uses the Second). This is Equation (5.12) in Adkins [1]:

*dU* = *TdS* − *pdV* (1)

where *U* is the internal energy, *T* the temperature, *S* the entropy, *p* the pressure and *V* the volume. This is because the change in internal energy of a closed system (*dU*) is the heat added to the system (*TdS*, by the Second Law) minus the work done by the system (*pdV*). Since *T* and *p* are intensive, and *dU* and *dV* are extensive, then *dS* is also extensive.

The trouble is that Equation (1) is strictly true only for closed systems in which *p* and *T* are well defined. For many simple systems this is true enough for practical purposes. Simple discussions also always consider the idealisation of “reversible” (or “quasi-static”) systems.

Jennifer Coopersmith’s magnificent book *Energy*, *the subtle concept* (2010) [12] describes the tortuous history of the discovery of the laws of thermodynamics over the past 400 years. She describes a wonderful cornucopia of steam engines, including the Marquis of Worcester’s “water-commanding engine” of 1655, Thomas Savery’s Steam Engine of 1698, Newcomen’s “fire engine” of 1717, James Watt’s condensing steam engines of the late eighteenth century, and Richard Trevithick’s high-pressure steam engines of the early 19th century, each of these representing a major innovation and improvement in performance beyond the earlier exemplars.

But it was not until the mid-19th century that our modern understanding of the science underlying these steam engines was established. The neologisms “*energy*” and “*thermodynamics*” were coined by William Thomson and the *concept* of entropy itself was described by Rudolf Clausius, all around 1850 (although Clausius only explicitly named “*entropy*” in 1865; Thomson was elevated to “Lord Kelvin” in 1892 and the absolute “Kelvin” scale of temperature that he proposed in 1848 was adopted as the S.I. measure in 1954).

**Figure 1:**
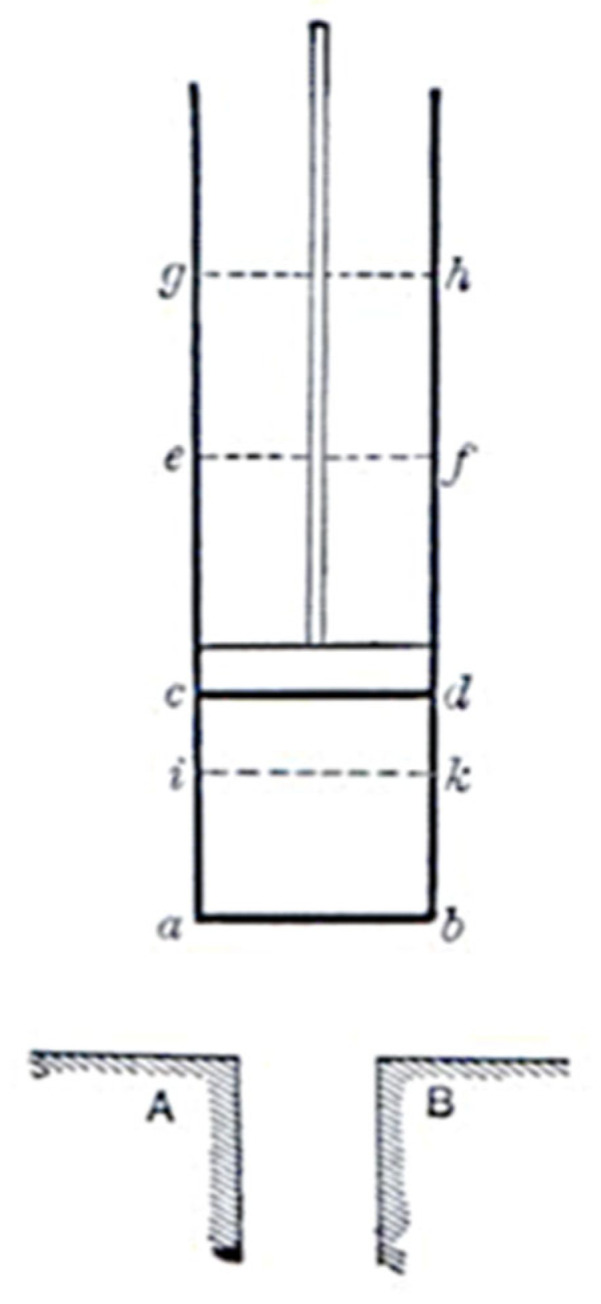
The “Carnot cycle”, from *Reflections on the Motive Power of Fire* (Sadi Carnot, 1824 [13], reproduced from Figure 1 (a facsimile) of the 1960 Dover reprint, and also reproduced by Jennifer Coopersmith: Figure 12.1 of *Energy*, *the subtle concept*, 2010, [12]).

Figure 1 shows Sadi Carnot’s 1824 [13] diagram for his eponymous “cycle”, still used today to teach physics students about thermodynamic efficiency (somewhat anachronistically since Carnot then still believed in *caloric*). The point is that all this 19th-century discussion of “thermodynamics” (Thomson’s word) was in terms of steam engines, with their cylindrical pistons (schematically indicated in Figure 1). But cylindrical geometry (embodying the technological necessities of practical steam engines) implicitly builds in (approximate) extensivity, and greatly simplifies the treatment while also being pedagogically intuitive.

## 3. Entropy Is Not Extensive in General

Jaynes ([2]) states that Pauli (1973 [14]) “*noticed [the] incompleteness of* [Clausius’ definition of entropy, which assumed a fixed system size] *and saw that if we wish entropy to be extensive*, *then that is logically an additional condition*, *that we must impose separately*”, and comments that the “*phenomenology … of thermodynamics … [makes] no reference to microstates [*etc.*] … As Helmholtz and Planck stressed*, *[thermodynamics] has a validity and usefulness quite independent of whether atoms and microstates exist*”. And Pauli determined the conditions required for entropy to be extensive.

But Parker & Jeynes (2019 [15]), in a full Lagrangian treatment using the Quantitative Geometrical Thermodynamics (QGT) formalism, have shown that the entropic and kinematical descriptions of a system are isomorphic (see their Table 1 for a summary), and have developed the treatment (see Equation (21) *passim* of Parker & Jeynes, 2021 [16]) to prove that *energy* is isomorphic to *entropy production* (since both *entropy production* and *energy* are Noether-conserved, as shown by their respective Euler–Lagrange variational descriptions). Note that entropy is *not* isomorphic to energy! Moreover, a fully complexified treatment shows that *energy* can be represented as effectively equivalent to *entropy production* (see Equation (23c) of Parker & Jeynes 2023 [17], although Kyriaki Aslani [18] has shown that the “complexification” can also arise from an even more general multivector geometric algebra framework).

The entropy *S* is given by an integral over the entropy production Π:

(2) S = ∫ Π dt

and therefore, since Π *is* extensive, it is only *if* the “constant of integration” is zero, trivially, that *S* is also extensive. Following Pauli [14], Jaynes [2] points out that this “constant” is actually a *function* in the general case, in which case *S* will be extensive only if this function is itself also extensive.

The distinction between *conservation* and *extensivity* should also be underlined. Conserved quantities (such as energy) are extensive, but extensive quantities (such as kinetic energy) are not necessarily conserved. A property is conserved if it conforms to a Euler–Lagrange variational description according to Noether’s first theorem. And Noether’s theorem concerns the geometrical (both temporal and spatial) symmetries of a system, such that the conserved property in question will also be additive (either temporally or spatially), thereby also being extensive. It is *entropy production* that is conserved (Equation (21) of [16], which cites Equation (13b) of [15]), but its temporal integration (entropy) is *not* conserved in the general case (and is therefore not necessarily extensive).

The entropy production is a conserved quantity since the *entropic* Euler–Lagrange equations are satisfied (these are given in terms of the *entropic* Lagrangian *L*_*S*_, for which see Equation (13a) of [15]):

(3) $$\frac{d}{dx_{3}}\left(\frac{\partial L_{S}}{\partial q_{n}^{\prime }}\right)-\frac{\partial L_{S}}{\partial q_{n}}\;=\;0\;(n\in \{1,2,3\})$$

where *q*′ and *q* are conjugate variables (in hyperbolic 3-space) of the system considered, and where the system has a symmetry direction along the *x*_3_ axis (where *x*_3_ is given in Euclidean space). This formalism of Quantitative Geometrical Thermodynamics (QGT) is described (and justified) in detail by Parker & Jeynes, 2019 [15]. Note that a Lagrangian is usually specified in terms with respect to time (d/d*t*) but QGT specifies the *entropic* Lagrangian in terms of geometry (d/d*x*_3_). The validity of this entropic Lagrangian is demonstrated by proving explicitly that the appropriate Euler–Lagrange equations are satisfied (Appendix C of [15]). This treatment permits *entropic* analyses both of stable entities (including DNA [15], Buckminsterfullerene [19], alpha particles [20]), and of the lifetimes of unstable ones (including the free neutron [21]).

It seems clear that *L*_*S*_ as used in Equation (3) may not be the most general expression of the idea, since this Lagrangian was set up to describe certain (simple) systems with well-defined symmetries (that is, where the *x*_3_ axis exists). As it stands, the QGT formalism (based on the complex analysis used in [17]) arises as a particular case from a more general analysis [18], which offers a wider approach to the issues of reversibility, irreversibility and symmetry in dynamic systems.

## 4. The Holographic Principle

The Holographic Principle was first explicitly defined by Gerard ‘t Hooft in 1993 and Leonard Susskind in 1995 [22], authoritatively reviewed by Rafael Bousso in 2002 [23] and helpfully summarised by Jacob Bekenstein in 2003 [24]: “*a fully three-dimensional [image of the Universe] could be [written] on a … surface*”. As such, its ramifications for entropy extend beyond Jaynes’ 1992 review of the field [2] (that we have copiously quoted from) and as a fundamental thermodynamics principle it was also unavailable to all the earlier noted scholars. It is in this light that the clarification of the (non) extensivity of entropy also becomes rather more obvious.

Parker & Jeynes (2021 [25]) have shown using QGT that the Holographic Principle is a consequence of the *entropic* Liouville Theorem (also obtaining the Bekenstein–Hawking expression for the entropy of a black hole as a special case; see their Equation (12a)). Parker et al. (2022 [20]) have used this result to directly obtain (ab initio, without any quantum mechanics) the matter radii of a variety of nuclei, including the alpha particle, which they show is a “unitary entity” (than which exists nothing simpler; such entities are reviewed by Jeynes & Parker, 2025 [26]).

The Holographic Principle entails that entropy *cannot* be extensive since it is proportional to the surface area of the enclosed volume. But although volume is extensive (for Euclidean space), surface area is not! However, the surface area of cylindrical volumes is *approximately extensive* (if the ends of the cylinders can be ignored). Thus, from a practical point of view, we can see how an idealised treatment based on the pistons of steam engines (ubiquitous in the 19th century) might leap to the conclusion that entropy (admittedly a rather abstract concept) was extensive. After all, energy is extensive and the entropy of a system is closely related to its energy. Note that Clausius intentionally chose his neologism “entropy” because of its phonetic resemblance to the word “energy”; however, the fundamental *physical differences* between (as opposed to resemblances of) these two apparently similar thermodynamical quantities need to be carefully taken into account.

It is interesting to note that one area of active current scientific interest is the coalescence of two black holes (BHs); and here, the question is whether the entropy of the resulting BH equals the sum of the entropies of the two initial BHs. The Holographic Principle (first derived in the context of black hole entropy) states that the BH entropy is proportional to the surface area (and is given by the Bekenstein–Hawking expression). That is to say, the entropy of the composite BH system is *less* than the sum of the individual BH entropies, in accordance with Jaynes’ 1992 statement [2] we quoted above. Clearly, the Holographic Principle guarantees that in general entropy is neither conserved nor extensive.

## 5. Relation of Entropy and Action

There is a strict isomorphism between the kinematic Principle of Least Action (PLA) (δ∫*L*d*t* = 0, where *L* is the appropriate Lagrangian and *t* is time as usual) and the entropic Principle of Least Exertion (PLE) (δ∫*L*_S_d*x*_3_ = 0, where *L*_S_ is the appropriate *entropic* Lagrangian and *x*_3_ is the appropriate geometrical axis of symmetry). But where action is a temporal line integral over the Lagrangian (∫*L*d*t*), entropy is (proportional to) a spatial line integral over the (entropic) Hamiltonian (∫*H*_S_d*x*_3_). For these relations, see Table 1 of Parker & Jeynes 2019 [15]; of course, the Hamiltonian and the Lagrangian are related through the Legendre transformation.

On the PLE (see Equation (13b) and Appendix C Equation (C.22) of [15]), Parker & Jeynes (2023 [27]) have shown that *exertion* is proportional to Jaynes’ *caliber* [28], which has proved a very useful concept (reviewed by Dixit et al., 2018 [29] and Martyushev, 2021 [5]). We should add that the “*Principle of Least Effort*” is now well known, first expressed by Guillaume Ferrero in 1894, but elaborated by George Zipf (1949 [30]). The idea was later developed in information science (see for example Herbert Poole, 1985 [31]), but these studies do not explicitly define a canonical Lagrangian (with conjugate variables, etc.) amenable to a variational analysis (as exhibited by the analytical Equation (3) above).

Frank Wilczek says (rather cautiously, 2015 [32]), “*A strong formal connection between entropy and action arises through the Euclidean*, *imaginary-time path integral formulation of partition functions. Indeed*, *in that framework the expectation value of the Euclideanized action essentially is the entropy*”. The complex time plane is necessarily invoked here, explicitly demonstrating the relation between entropy and action (e.g., see [17]), the mathematical transformation described by Wilczek being the Wick rotation, which is often considered only to be a mathematical trick employed to calculate a finite integral (that converges, but which would otherwise oscillate). However, the Wick rotation indicates the presence of some deep and interesting physics relating entropy and action.

To that end, J.-P. Badiali [33] elaborates on this relation between entropy and action; his Equation (3) is

δ*S*/*k*_B_ = −δ*A*^E^/*ħ* (4)

where *S* is the entropy and *A*^E^ is the “Euclidean action” (and *k*_B_ and *ħ* are Boltzmann’s constant and the reduced Planck constant, as usual). This is a (rather crude) approximation to the Cauchy–Riemann relations given by Parker & Jeynes 2023 [17] (their Equation (5)). The “Euclidean action” of Equation (4) here refers to the Wick rotation (from Minkowski spacetime to a Euclidean spacetime with “real” time) applied in quantum field theory to any of the Lagrangian and Hamiltonian actions in the relevant path integral.

The extensivity of the physical quantity *action* is generally assumed, especially where two independent and separable systems are under consideration. Specifically, the action functional *S*(*t*), calculated over time, has the strong appearance of extensivity in time since the “system” can be split into two temporally successive “sub-systems” which are clearly additive (however, note that this temporal additivity also requires the overall system be local):

(5) $$S\left[x\left(t\right)\right]\;=\;\int_{t_{i}}^{t_{f}}L\left(x,\dot{x},t\right)dt\;=\;\int_{t_{i}}^{t_{1}}Ldt+\int_{t_{1}}^{t_{f}}Ldt\;=\;S_{1}+S_{2}$$

where *L* is the appropriate Lagrangian. Moreover, when considering an overall system with two independent degrees of freedom *x* and *y*, say, and using the path integral formalism (see for example Equation (10) of Feynman, 1948 [34]) for the evolution of its (quantum mechanical) wavefunction, we can write

(6) $$\int \int DxDye^{\frac{i}{\hbar }\left(S_{x}+S_{y}\right)}=\left(\int Dxe^{\frac{i}{\hbar }S_{x}}\right)\left(\int Dye^{\frac{i}{\hbar }S_{y}}\right)$$

where D*x*, D*y* denote functional integrals. The factorisation of Equation (6) also apparently indicates the extensivity of the action. (Note that the extensivity here is expressed through the additivity of the phases within the arguments of the complex exponentials forming the integrals.)

However, the caveats are clear: For an extensive *action* of the system, its subsystems must be *independent* of each other (any coupling terms surrender extensivity) and temporal additivity requires the overall system be *local*. It is also clear that where boundary or topological terms dominate in the descriptions of the subsystems, this is at the expense of extensivity. Thus, when the systems are no longer separable, additivity is no longer applicable and action extensivity fails.

These same caveats also apply to the issue of the extensivity of entropy: both independent subsystems and spatial additivity are required (the latter is largely satisfied by cylindrical geometries as previously noted, and see Equation (10b) below), and boundary or topological terms must be insignificant (in this case the circular end-face areas of the cylinders are neglected).

Extensivity also requires locality; however, it is now well known that the Universe has strongly non-local attributes since quantum mechanics requires non-locality: quantum non-locality is now very well established (initially by Aspect et al. 1982 [35], recently by Giustina et al., 2015 [36] and Proietti et al., 2019 [37]) and has been discussed by Tim Maudlin (2010 [38]).

## 6. Non-Extensive Statistical Mechanics

It is widely thought that *thermodynamics* can be “reduced” to *statistical mechanics*—that is, that statistical mechanics is “more fundamental” than thermodynamics. And indeed, in the “thermodynamic limit” (of large numbers), classical thermodynamics can properly be thought to “emerge” from statistical mechanics. This is an example of what Michael Berry calls a “*Singular Limit*” [39]: when one representation of reality (in this case the concepts associated with “classical thermodynamics” considerations) “emerges” from a (supposedly more “fundamental”) representation (“statistical mechanics”) through some limiting process (in this case letting the number of particles in the system get very large). In this context it is known that critical thermodynamical transitions have fractal properties (see for example Bernard Halperin 2019 [40], and Yonglong Ding 2024 [41]); that is, certain thermodynamic parameters are not well defined at those transitions. This view of “emergence” is described in Robert Batterman’s 2002 monograph [42]. Similar to the considerations of classical thermodynamics, such statistical mechanical descriptions are also often thought to be extensive; but studies of *non-extensive* statistical mechanical systems have also begun to be studied in recent decades.

Tsallis entropy [43] is an explicitly non-additive generalisation of Boltzmann–Gibbs (or Shannon) entropy that finds practical applications in situations featuring long-range interactions and correlations, or heavy-tailed (or power-law) distributions, where the non-locality of these systems is therefore (unsurprisingly) also implicitly indicated. Note that the B-G entropy is predicated on *independent* microstates and the Shannon entropy is predicated on *independent* items of “information” (that is, Shannon information, which is shorn of any semantic content), all of which suggest a degree of additivity (as discussed above). The point is that there also manifestly exist what Tsallis calls “*non-additive universality classes*” [44] into which we can sort various types of physical systems. That is to say, it is well known that these other classes represent intrinsically non-extensive entropic measures, in contrast to the case of B-G entropy, which in general is also non-extensive (although often assumed to be extensive).

Rényi entropy [45] is another non-additive generalisation, used originally to give alternative proofs of (for example) the central limit theorem in entirely information-theoretic terms. It has also been used by Ansari & Nazarov (2015) [46] to calculate the entropy production of generic (ideal) quantum heat engines. The rationale for this is that the Rényi entropy production naturally separates into coherent and incoherent parts. However, despite these formal advantages Ansari & Nazarov note that technical problems remain (the quantum entropy production “*requires revision and clarification*”).

Other workers have developed a variational approach to handle complex cases that are manifestly non-additive. Pachter et al. point out, in their review of the *Maximum Caliber* principle (2024 [47]), that maximum entropy methods yield probability distributions with exponential tails. Note that Parker & Jeynes, 2019 [15], independently demonstrated the existence and some properties of the *Maximum Exertion* principle, where *exertion* is directly proportional to Jaynes’ *caliber* [27].

Small-system thermodynamics is required for handling many real situations, and Terrell Hill’s (1962 [48]) seminal introduction to this has led the way both to the fluctuation theorems (first introduced by Evans et al. in 1993 [49]) and to the application of “Maximum Entropy” methods (for example Perushottam Dixit, 2013 [50]) which demonstrate (again) precisely the physical reality of heavy-tailed probability distributions characteristic of non-additive entropies. Terrell Hill’s treatment may also be applied to large systems with long-range interactions: Campa et al. (2018 [51]) explicitly point out that systems with such interactions are common (including self-gravitation, plasmas, or fluid dynamics).

de Miguel & Rubí (2020 [52]) explicitly treat the “non-extensive thermodynamics” of small systems, where their Hamiltonian approach to “thermodynamics at strong coupling” (TSC) is different in kind to Hill’s heuristic approach to classical thermodynamics.

Chamberlin & Lindsay’s review (2024 [53]) gives a valuable overview of how to handle the fluctuation theorems in real systems, starting with Hill’s “small-system thermodynamics” and pointing out that at maximum entropy, statistically independent subsystems may occur in systems of any size. They also underline that a generalised ensemble can be defined that is completely open, having *no* extensive environmental variables (Chamberlin et al., 2021 [54]).

An alternative approach to the study of “small-system thermodynamics”, but from a non-statistical mechanical perspective, is found in Quantitative Geometrical Thermodynamics (QGT [15]), which considers the physics that equally applies to *small* systems (to which the fluctuation theorems apply: see Bustamante et al. 2005 [55]) but from a *geometrical* point of view. Edwin Jaynes has long asserted that, properly, both “Boltzmann entropy” and “Shannon entropy” are *entropy*, even though Shannon entropy (and QGT) and Boltzmann entropy are handled in formally entirely different ways. Where the latter analyses the (statistical) behaviour of *ensembles*, the former can be represented as concentrating on the (holistic) properties of *unitary entities* (see [26]), in which case geometrical considerations, like the Holographic Principle or Liouville’s Theorem, become important (see [25]). QGT automatically applies to small systems, and it entirely avoids the issue of singular limits (and emergence) since no limiting processes are implied. This is consistent with the view of Michael te Vrügt et al. [8], who show that even for large systems thermodynamics can only be reduced to statistical mechanics by a manipulation involving the *coarse-graining* required to specify the partition function; they do this by a formal demonstration that the “master equation” (the *irreversible* transport equation of thermodynamics; see their Equation (8.3)) is derived from the exact (*reversible*) transport equation of statistical mechanics (their Equation (8.2)), only with the addition of *two* assumptions—the Markov approximation, and the past hypothesis—both of which they discuss at length. Taken together, we see here that geometrical considerations (as opposed to statistical mechanical ones) can also lead to thermodynamic quantities, the key point being that the entropy measures in both are *not* intrinsically extensive in nature.

## 7. Non-Extensivity of Entropy in QGT

Quantitative Geometrical Thermodynamics (QGT) describes entropy using the symplectic geometry familiar from classical Hamiltonian mechanics. That is to say, conjugate *entropic* parameters {*p*,*q*} (being respectively *entropic* momentum and *hyperbolic* space) are employed in the canonical relations *p*′ = ∂*L_S_*/∂*q* and *q*′ = −*L_S_*/∂*p*) where *L_S_*(*p*,*q*,*x*_3_) is the *entropic* Lagrangian, *x*_3_ is the (Euclidean space) axis of symmetry for the entropic geometry (as previously discussed), and the prime indicates differentiation with respect to *x*_3_. That is to say, QGT is based on a *spatial* calculus (with respect to *x*_3_), whereas the conventional mechanics of the PLA is based on a *temporal* calculus (with respect to time *t*). Of particular relevance is that the entropic hyperbolic space *q* is defined as (Equation (9a) of [15]):

(7) $$q\;=\;R\ln\frac{x}{R}$$

The logarithmic definition of *q* is what makes it hyperbolic, in contrast to the Euclidean space *x* (within the argument of the logarithm in Equation (7)). Here, *R* is a Euclidean length metric that defines the entropic (logarithmic) scale of interest. Previously (see for example Equations (6) and (12), etc., of [16]), we showed that the entropy production Π is a relativistic quantity in spacetime (just like energy–momentum in kinematics) and is relativistically related to the entropic momentum *p*. More specifically, the entropy production Π is simply related to the entropic Hamiltonian *H_S_* by Equation (13) of [16]:

(8) $$\Pi \;=\;cH_{S}$$

(where *c* is the speed of light as usual) such that the overall entropy *S* of a system is given by a (spatial) line integral over the entropic Hamiltonian (or equivalently the entropy production) over a circumferential spiral trajectory *l* around the system in Euclidean space:

(9) $$S\;=\;\int H_{S}dl\;=\;\frac{1}{c}\int \Pi dl$$

Note that for the special case of the system being described by a cylindrical geometry, the associated spiral trajectory is proportional to the longitudinal axis *x*_3_, such that *l* = *χx*_3_ (the *χ* being a constant of proportionality; see following Equation (12) of [15]), and we have

(10a) $$S=\int H_{S}dl=\frac{\chi }{c}\int \Pi dx_{3}$$

The additivity intrinsic to Equation (10a) (equivalent to that seen in Equation (5)) is obvious:

(10b) $$S\;=\;\frac{\chi }{c}\int_{x_{i}}^{x_{f}}\Pi dx_{3}\;=\;\int_{x_{i}}^{x_{1}}\Pi dx_{3}+\int_{x_{1}}^{x_{f}}\Pi dx_{3}\;=\;S_{1}+S_{2}$$

However, we emphasise again that the additivity here is only true for the special case of a cylindrical geometry, and we also note the other caveats already discussed in the previous section.

We now express Equation (2) in differential form, and apply it to Equation (9), in order to derive an expression which is *generally* true:

(11) $$\frac{dS}{dt}\;=\;\frac{d}{dt}\left[\frac{1}{c}\int \Pi dl\right]\;=\;\frac{1}{c}\int \left(\frac{dl}{dt}\right)d\Pi \;=\;\int d\Pi \;=\;\Pi$$

Note that because d*S*/d*t* ≡ Π we also have the identity *c* ≡ ∂*l*/∂*t*. That is to say, the integral summation of the infinitesimal entropy production dΠ (Equation (11)) indicates the intrinsic extensivity of the entropy production Π (since extensivity *means* additivity). The extensivity of the entropy production as seen on the RHS of Equation (11) is fundamental, and, indeed, consistent with the conservation of entropy production as per Noether’s theorem (see further Equation (23c) *passim* of [17]).

We can also see this from consideration of the entropic momentum (Equation (9b) of [15] and Equation (9a) of [25]):

*p* ≡ *m*_*S*_*q*′* (12)

where *m_S_* is the entropic mass, and *q*′* is the phase hyperbolic velocity (where *q*′*∙*q*′ ≡ 1; Equation (23b) of [25]). Therefore, the entropic momentum is entirely isomorphic to the conventional kinematic momentum, both momenta being products of the (entropic) mass and the (entropic, hyperbolic) velocity. Explicitly differentiating Equation (7) with respect to the axial symmetry direction *x*_3_ (noting that *q*′ is *defined* as *q*′ ≡ ∂*q*/∂*x*_3_), we see that the phase hyperbolic velocity *q*′* is given by

(13) $$q^{\prime *}\equiv {\left[\frac{\partial q}{\partial x_{3}}\right]}^{-1}={\left[\frac{\partial }{\partial x_{3}}\left(R\ln\frac{x}{R}\right)\right]}^{-1}={\left[\frac{R}{x}\frac{\partial x}{\partial x_{3}}\right]}^{-1}=\frac{x/R}{\partial x/\partial x_{3}}$$

and from Equation (12), multiplying Equation (13) by the entropic mass *m_S_* yields the entropic momentum *p*. The key thing to note is that the entropic momentum *p* is related to the entropy production Π in exactly the same relativistic way that the (kinematical) energy and momentum are related (see Equation (13) of [16]). That is to say, just as entropy production is a conserved and extensive quantity, so is the entropic momentum. This can be seen from combining Equations (12) and (13), and assuming ∂*x*/∂*x*_3_ = *C* can be considered constant (which is approximately true for any system geometry where an appropriate longitudinal *x*_3_ axis can be identified), such that

(14) $$p\equiv m_{S}q^{\prime *}=x\frac{m_{S}}{RC}$$

in which case the properties of the entropic momentum are determined by the spatial quantity *x*, which is additive, being Euclidean. It is therefore clear that the entropic momentum is additive (extensive) just like its kinematic counterpart. It is interesting to note that whereas the hyperbolic spatial quantity *q* is *not* additive (being hyperbolic and non-Euclidean in nature), the entropic momentum (which is essentially based on the spatial gradient of *q*) *is* additive.

This can be simply understood (in contrast to the hyperbolic quantity *q* which is intrinsically non-additive and also non-local) from the fact that the hyperbolic velocity *q*′* is a purely *local* quantity as per Equation (13) (it being the *local* spatial gradient) and indeed is therefore also Euclidean in nature. Note that hyperbolic space can be considered *locally* to be Euclidean: the non-additivity of *q* only becomes particularly evident when considered over larger distances.

Thus, entropy playing itself out in (entropic) hyperbolic *q* space is non-local (indeed holographic) and is therefore non-extensive, whereas the (extensive) entropy production is related relativistically to the (extensive) entropic momentum (see Equations (8) and (13) of [16]) and both (being described in Euclidean space) are as extensive (and additive) as are energy and kinematic momentum.

## 8. Non-Extensivity of Information

Following Claude Shannon’s seminal *A Mathematical Theory of Communication* (1948), Léon Brillouin popularised the idea of “negentropy” [56] as being equivalent to “Shannon information” (Brillouin abbreviated Erwin Schrödinger’s “negative entropy”, introduced in 1944 [4]), particularly when considered through the perspective of noise (i.e., the most informative signal is practically indistinguishable from noise; and a “high entropy” signal is also essentially noisy; see Hermann Haus, 2000 [57]). That is, information and entropy essentially have the same physical properties, both being intrinsically acausal (unpredictable and non-deterministic). Parker & Jeynes [15] also showed that Shannon information and entropy are in fact well represented geometrically as Hodge duals, allowing a holographic function “info-entropy” to be formed (see their Equation (4) *passim*, including their discussion of meromorphic functions). Negentropy was discussed very recently by Didier Lairez [58].

Note that “Shannon information” (defined by the communications engineers) includes only the *syntactical* elements of the information: it explicitly *excludes* the semantic elements (the *meaning*!), which is what we need our *information* to have. But “Shannon information” and “entropy” are entirely commensurate quantities (also proved recently by Parker et al. [59]), so that *information* will be as little (or as much) extensive as is *entropy* itself. That is, since entropy is not extensive in general, neither can be information.

Similarly, quantum mechanics generally assumes that unitary Hamiltonian evolution implies the conservation of information. However, this would only be true were the geometry of the Universe Euclidean, local, and non-holographic. Yet, contemporary physics is instead indicating the opposite. Since (Shannon) information is not extensive in general, neither can it be conserved (in general). That is to say, it is the information production (i.e., rate of change of information) that can be expected to exhibit conservation properties akin to those also associated with entropy production.

Although it is simple to contrive examples where information is additive and appears conserved (just as for the entropy of cylindrical pistons, and the local evolution of a system’s action, as discussed above), *in general* it is clear that, just like its physical equivalent entropy, information is a non-extensive quantity.

## 9. Discussion

Idealising cylinders led to a treatment in which entropy could be treated as extensive, a rather good approximation given the experimental precision available in the 19th century, and the rather abstract character associated with entropy ever since its first articulation. Moreover, since much of the experimental work in the nineteenth century would have been performed using piston cylinders and steam engines (and even Joule’s seminal work on the transformation of work into heat using a paddle was undertaken within a cylindrical barrel) there would have been quiet satisfaction about the (apparent) “experimental confirmation” that entropy is indeed extensive.

In any case, space is probably hyperbolic (not Euclidean; see §2.7 of Penrose, 2004 [60], and Parker et al. 2025 [61] on hyperbolic probability), so that even regarding volume as “extensive” is strictly an approximation, however good it might be on the scales we are usually interested in, indeed for the same reasons that Newtonian mechanics (Euclidean metric) is such a good approximation at low velocities to the more accurate relativistic measures (hyperbolic metric).

In general, one suspects that the categories *intensive*/*extensive* are themselves (very useful) idealisations: that is, any thoroughly realistic treatment would expose them as approximations, strictly speaking.

It is clear that apparent “proofs” of the extensivity of entropy have implicitly entailed any example system conforming to various fortuitous (if indeed subtly intuitive) idealisations (such as having a geometry appropriate to a cylindrical structure so that it is spatially additive, allowing one to ignore boundary constraints such as the end-faces of the cylinder). Also entailed would be the assumption that the Universe is essentially *local* in character (implying that the Holographic Principle is not universally valid). Indeed, for entropy to be extensive implies a Universe that is significantly different to what modern science is now revealing to us: non-local, hyperbolic, and holographic in nature.

Releasing science from the assumption that entropy is extensive will perhaps enable contemporary physics to be able to properly embrace, assimilate and then develop many of the most important scientific discoveries about the Universe that have been made in recent years, and indeed overcome some of the many paradoxes now currently evident about the cosmos. It is well known that thermodynamics and entropy are foundational to many of the most important physical processes occurring in the Universe and our scientific descriptions of reality, yet falsely premising the extensivity of entropy will inevitably corrupt ideas about the fundamental nature of the Universe.

## 10. Conclusions

Probably, the categories *intensive* and *extensive* are idealisations of reality (and therefore only ever approximate, strictly speaking). For example, even volume is only strictly *extensive* in Euclidean space, yet space is almost certainly not Euclidean. Moreover, temporal additivity (as in Equation (5)) requires the overall system be local, but the Universe is *not* local (the non-locality of quantum mechanics is now very well established).

In any case, a Quantitative Geometrical Thermodynamics (QGT) formalism makes it clear that *energy* is isomorphic to *entropy production*, and *entropy* itself is closely related to the *action*. Both *energy* and *entropy production* are Noether-conserved (and therefore extensive, in an idealised description of reality), but neither *entropy* nor *action* are. Moreover, the Holographic Principle is shown by QGT to be general; it is also incompatible with entropy being extensive.

Formally, since the entropy is an integral over time of the (extensive) entropy production, entropy can be extensive only if the constant function of integration is also extensive (or, trivially, zero). There are some systems where this is (approximately) true. But these are mostly rather simple systems (useful for superficially justifying the assertion that “entropy is extensive”). But in general entropy is not extensive.

It is clear that more than 150 years after its initial “discovery” entropy is continuing to surprise scientists with its essential character and properties, and is leading to new insights into the meaning of extensivity in science. In particular, it is clear that entropy is also strongly geometrical in character, and is also revealing important aspects of the physical structure of the Universe and the nature of reality.

## Figures and Tables

**Table 1:** Some conjugate pairs of thermodynamic variables (after Table 1.1 of C. J. Adkins’ classical textbook *Equilibrium Thermodynamics* [1], explicitly representing *entropy* as extensive).

System	Conjugate Variables	Intensive Variable	Extensive Variable
Fluid	*p*, *V*	pressure	Volume
Wire	*F*, *L*	torsional force	Length
Film	*γ*, *A*	surface tension	Area
Magnetic material	*H*, *M_m_*	magnetic field	Magnetic dipole moment
Dielectric	*E*, *M_e_*	electric field	Electric dipole moment
All systems	*T*, *S*	temperature	Entropy
Generalised	*X*, *x*	force	Displacement

## Data Availability

The original contributions presented in this study are included in the article. Further inquiries can be directed to the corresponding author.
